# Immunoinformatics Approach for Multiepitopes Vaccine Prediction against Glycoprotein B of Avian Infectious Laryngotracheitis Virus

**DOI:** 10.1155/2019/1270485

**Published:** 2019-03-18

**Authors:** Sumaia A. Ali, Yassir A. Almofti, Khoubieb A. Abd-elrahman

**Affiliations:** ^1^Department of Molecular Biology and Bioinformatics, College of Veterinary Medicine, University of Bahri, Khartoum, Sudan; ^2^Department of Veterinary Medicine and Surgery, College of Veterinary Medicine, Sudan University of Science and Technology, Sudan; ^3^Department of Pharmaceutical Technology, College of Pharmacy, University of Medical Science and Technology (MUST), Khartoum, Sudan

## Abstract

Infectious laryngotracheitis virus (ILTV) is a* gallid herpesvirus type 1*, a member of the genus* Iltovirus*. It causes an infection in the upper respiratory tract mainly trachea which results in significant economic losses in the poultry industry worldwide. Vaccination against ILTV produced latent infected carriers' birds, which become a source of virus transmission to nonvaccinated flocks. Thus this study aimed to design safe multiepitopes vaccine against glycoprotein B of ILT virus using immunoinformatic tools. Forty-four sequences of complete envelope glycoprotein B were retrieved from GenBank of National Center for Biotechnology Information (NCBI) and aligned for conservancy by multiple sequence alignment (MSA). Immune Epitope Database (IEDB) analysis resources were used to predict and analyze candidate epitopes that could act as a promising peptide vaccine. For B cell epitopes, thirty-one linear epitopes were predicted using Bepipred. However eight epitopes were found to be on both surface and antigenic epitopes using Emini surface accessibility and antigenicity, respectively. Three epitopes (_*190*_*KKLP*_*193*_, _*386*_*YSSTHVRS*_*393*_, and _*317*_*KESV*_*320*_) were proposed as B cell epitopes. For T cells several epitopes were interacted with MHC class I with high affinity and specificity, but the best recognized epitopes were _118_*YVFNVTLYY*_*126*_, _335_*VSYKNSYHF*_*343*_, and _*622*_*YLLYEDYTF*_*630*_. MHC-II binding epitopes, _301_*FLTDEQFTI*_*309*_,_*277*_*FLEIANYQV*_*285*_, and _*743*_*IASFLSNPF*_*751*_, were proposed as promising epitopes due to their high affinity for MHC-II molecules. Moreover the docked ligand epitopes from MHC-1 molecule exhibited high binding affinity with the receptors; BF chicken alleles (BF2 2101 and 0401) expressed by the lower global energy of the molecules. In this study nine epitopes were predicted as promising vaccine candidate against ILTV.* In vivo *and* in vitro *studies are required to support the effectiveness of these predicted epitopes as a multipeptide vaccine through clinical trials.

## 1. Introduction

Infectious laryngotracheitis (ILT) is classified as a* gallid herpesvirus 1* which belongs to the fay Herpesviridae, genus Iltovirus [1–3]. The virus is included within List E of the Office International des Epizooties (OIE). It causes a major viral respiratory disease of chicken [2]. The disease causes marked economic losses of poultry industry with mortality reaching 70%, especially in high- density poultry-producing regions [3]. High mortality, demonstrated by the severe form of the disease, is a result of severe tracheal lesions in the respiratory tract, significant respiratory distress, expectoration of bloody sputum, sneezing and persistent nasal discharge decreased egg production, weight loss, and susceptibility to infections with other pathogens [4]. The mild form exhibited low mortality, mucoid tracheitis, and sinusitis [5, 6].

Vaccination against the viral diseases is very important for protection, due to the lack of appropriate antiviral drugs, high cost, and time consuming of development of new antiviral drugs. Different types of vaccines are available for ILTV such as vaccines produced in chicken-embryo-origin (CEO), tissue-culture-origin (TCO), and recombinant vaccines [5]. However, vaccination against ILTV is recommended only in endemic areas to prevent transmission of the virus by latent infected carriers' birds to nonvaccinated healthy flocks [1, 7, 8]. Moreover current vaccines are themselves mildly pathogenic and modified live ILT vaccines increase the virulence of the disease by mutation during bird-to-bird passage in the field [2, 9, 10]. DNA encoding glycoprotein B vaccine was found to give levels of protection when given intramuscularly comparable to traditional live-attenuated ILTV vaccines. Glycoprotein B and D genes of ILTV have been used to produce immunogenic proteins to elicit protective immune response. These glycoproteins which are located on the viral envelope and the surface of infected cells are required for viral attachment [1]. Developing of new drugs to treat viral diseases is very expensive and time consuming. Therefore, vaccines remain the best choice to protect animals and humans from viruses and other pathogens. In addition traditional techniques of live-attenuated or inactivated vaccines have the risk of allergic reactions. Peptide vaccines are economically reasonable, require less time for development, and hold the promise of multivalent dosages [11–13]. Recently, bioinformatics software has been used largely to design synthetic peptide vaccines, based on B and T cell responses [14]. The design of multipeptide vaccines using computational model that links various immunoinformatic prediction tools is known to produce satisfactory results [15, 16]. The safety, accuracy, feasibility, and speed of these vaccines were well discussed through various computational studies [17, 18]. Thus, it is essential to design safe effective vaccine against ILTV that prevents birds from being carriers of the disease using bioinformatics tools. The aim of the present study was to design a vaccine for ILT virus using peptides predicted from glycoproteins especially type B as an immunogen to stimulate protective immune response. The reason of selecting glycoprotein B as a target is due to its function in host attachment and in stimulating immune response in the host.

## 2. Materials and Methods

### 2.1. Protein Sequence Retrieval and Phylogeny

Forty-four envelope glycoprotein B (GB) sequences of virulent isolates of ILTV were retrieved from GenBank of National Center for Biotechnology Information (NCBI) (http://www.ncbi.nlm.nih.gov/protein) in August 2017. The strains of the virus were isolated from chicken from different geographic regions. Complete sequences of all gene subtypes were selected for various immune-bioinformatics analysis. Retrieved strains and their accession numbers and geographical regions are listed in Table 1.

### 2.2. Phylogenetic Evolution

Phylogenetic tree of the retrieved sequences of glycoprotein B of ILTV was created using phylogeny.fr online software (http://phylogeny.lirmm.fr/phylo_cgi/index.cgi) [19].

### 2.3. Multiple Sequence Alignment

The retrieved sequences of ILTV glycoprotein B (GB) were subjected to multiple sequence alignments (MSA) to obtain the conserve regions. This was performed using BioEdit software version 7.2.5 with the aid of ClustalW as applied in the BioEdit program to construct the alignment [20].

### 2.4. Sequence-Based Method

The reference sequence (YP_182356.1) of ILT virus glycoprotein B (GB) was submitted to different prediction tools at the Immune Epitope Database analysis resource (http://www.iedb.org/). Epitope analysis resources were used to predict B and T cell epitopes [21]. Predicted epitopes were then investigated in aligned retrieved GB sequences after MSA for conservancy. Conserved epitopes would be considered as candidate epitopes for B and T cells.

#### 2.4.1. B Cell Epitope Prediction

**Figure 1:**
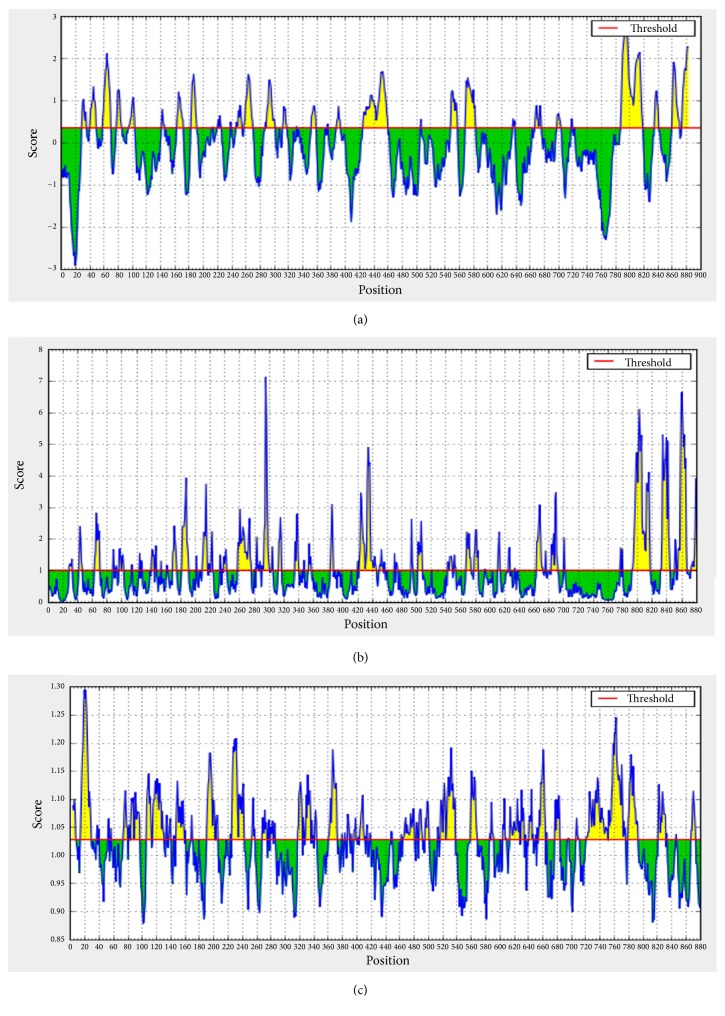
Prediction of B cell epitopes using (a) Bepipred linear epitope, (b) Emini surface accessibility, and (c) Kolaskar and Tongaonkar Antigenicity methods. Yellow areas above the threshold (red line) are suggested to be a part of B cell epitope, while green areas are not.

Identification of the surface accessibility, hydrophobicity, flexibility, and antigenicity was performed by analyzing candidate epitopes using several B cell prediction methods from Immune Epitope Database (http://tools.iedb.org/bcell/). BepiPred linear epitope prediction from Immune Epitope Database (http://tools.iedb.org/bcell/result/) was used to predict linear B cell epitopes with default threshold -.012 [22]. Emini surface accessibility prediction tool of IEDB was performed to detect the surface accessible epitopes with default threshold 1.000 [23], while the prediction of epitopes antigenicity sites of candidate epitopes was achieved to identify the antigenic sites using Kolaskar and Tongaonker antigenicity method (http://tools.immuneepitope.org/bcell/) with default threshold 1.027 [24]. The thresholds of these methods are demonstrated in Figure 1.

#### 2.4.2. T Cell Epitope Prediction

*(1) Cytotoxic T-Lymphocyte Epitopes Prediction and Interaction with MHC- I*. The major histocompatibility complex-1 (MHC class I) binding prediction tool (http://tools.iedb.org/mhci/) was used to predict Cytotoxic T cell epitopes [25]. Analysis was achieved using human HLA alleles, due to the lack of chicken alleles in IEDB data set. Artificial neural network (ANN) was used to predict the binding affinity [26, 27]. Peptide length for all selected epitopes was set to 9 amino acids (mers). The half-maximal inhibitory concentration (IC50) values required for the peptide's binding to the specific MHC-I molecules were set less than or equal to 300 nM.

*(2) Prediction of T Cell Epitopes and Interaction with MHC Class II*. The MHC class II binding prediction tool (http://tools.iedb.org/mhcii/) was used to predict T cell epitopes. IC50 for strong binding peptides was set less than 1000 to determine the interaction potentials of T cell epitopes and MHC class II allele (HLA DR, DP and DQ). Human MHC class I and II alleles were used in this study due to the difficulty to determine MHC B complex alleles in poultry. NN-align method was also used with IC50 less than or equal to 1000 nM [28]. Peptides with low IC50 value were proposed to be promising MHC-II epitopes.

### 2.5. Homology Modeling

#### 2.5.1. Structural Prediction of the Reference Sequence of ILTV Glycoprotein B

Homology modeling was used for constructing the three-dimensional (3D) structure of the reference sequence of ILTV glycoprotein B. Raptor X structure prediction server (http://raptorx.uchicago.edu/StructurePrediction/predict/) was used for this purpose. The 3D structure was then treated with Chimera software 1.8 to display the position of proposed epitopes [29–32].

#### 2.5.2. Structure of BF Chicken Alleles

Protein sequence and PDB ID of chicken alleles (BF2*∗*2101 & BF2*∗*0401) were retrieved from the NCBI database/ (PDB: 4D0C, CAK54661.1 and PDB: 4D0C CAK54660.1) and submitted to Raptor X server (http://raptorx.uchicago.edu/) for homology modeling. Chimera software was used to display 3D structure of BF alleles [29–32].

#### 2.5.3. Structure of Predicted Epitopes

The homology modeling of the MHCI predicted peptides was performed with PEP FOLD3 (http://bioserv.rpbs.univ-paris-diderot.fr/services/PEP-FOLD3/) to predict the linear structures from amino acid sequences [33–35].

### 2.6. Molecular Docking

Molecular docking was performed according to peptide-binding groove affinity, between chicken BF alleles (BF2*∗*2101 & BF2*∗*0401) and the proposed peptides from MHCI. Chicken BF alleles were set as receptors and the proposed peptides were set as ligands. Molecular docking technique of 3D structure of BF alleles and 3D modeled epitopes was performed using PatchDock online autodock tools; an automatic server for molecular docking (https://bioinfo3d.cs.tau.ac.il/PatchDock/) by submitting PDB of ligands and receptors after homology modeling by Raptor X server and PEP FOLD3 [36, 37]. Firedock was used to select the best models [38]. Visualization of the result was performed off-line using UCSF-Chimera visualization tool 1.8. [29].

## 3. Results and Discussion

In the vaccine industry, presenting a specific antigen or a host of antigens to the immune system is necessary to increase immunity against viral diseases. The functional component of the vaccine should be able to stimulate the immune system, by using vaccines containing intact inactive components (attenuated viruses; purified immunogenic parts of the pathogen) to trigger immune response [27, 39]. It is known that the use of whole viral proteins to induce an immune response is not necessarily but small portions of protein called antigenic determinants or adhesive epitopes can be used to stimulate the desired immunity [40].

The use of bioinformatics analyses is an applicable method for predicting and designing new multiepitope vaccines against animals' infectious diseases as well as chickens [17, 41, 42]. This is the first in silico study to design peptide vaccine against avian ILTV through humoral and cell mediated immune responses. The expected epitopes in this study could help in prevention of latent infection caused by the use of attenuated vaccines and developing more effective and trustable prophylactic and therapeutic vaccines than conventional methods.

### 3.1. Sequences Alignment

**Figure 2:**
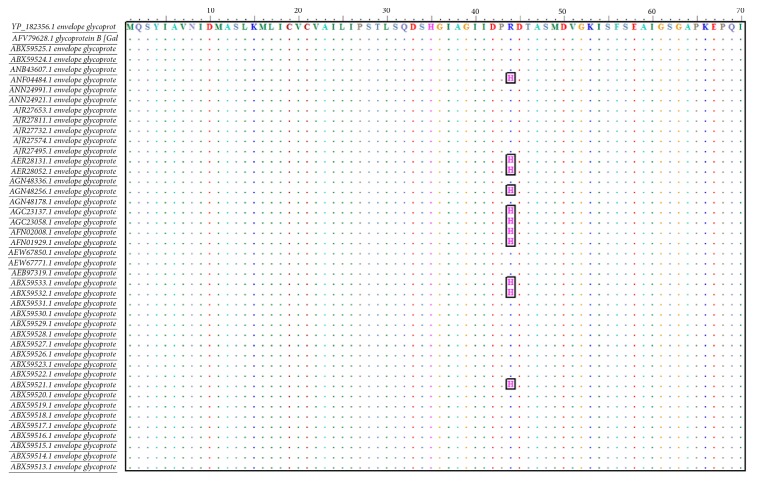
Multiple sequence alignment (MSA) of the retrieved strains using BioEdit software and ClustalW. Dots indicated the conservancy and letters in cubes showed the alteration in amino acid.

Alignment of all retrieved sequences using ClustalW through BioEdit software showed high conservancy between the aligned sequences. As shown in Figure 2, the conserved regions were recognized by identity and similarity of amino acid sequences.

### 3.2. Phylogenetic Evolution

**Figure 3:**
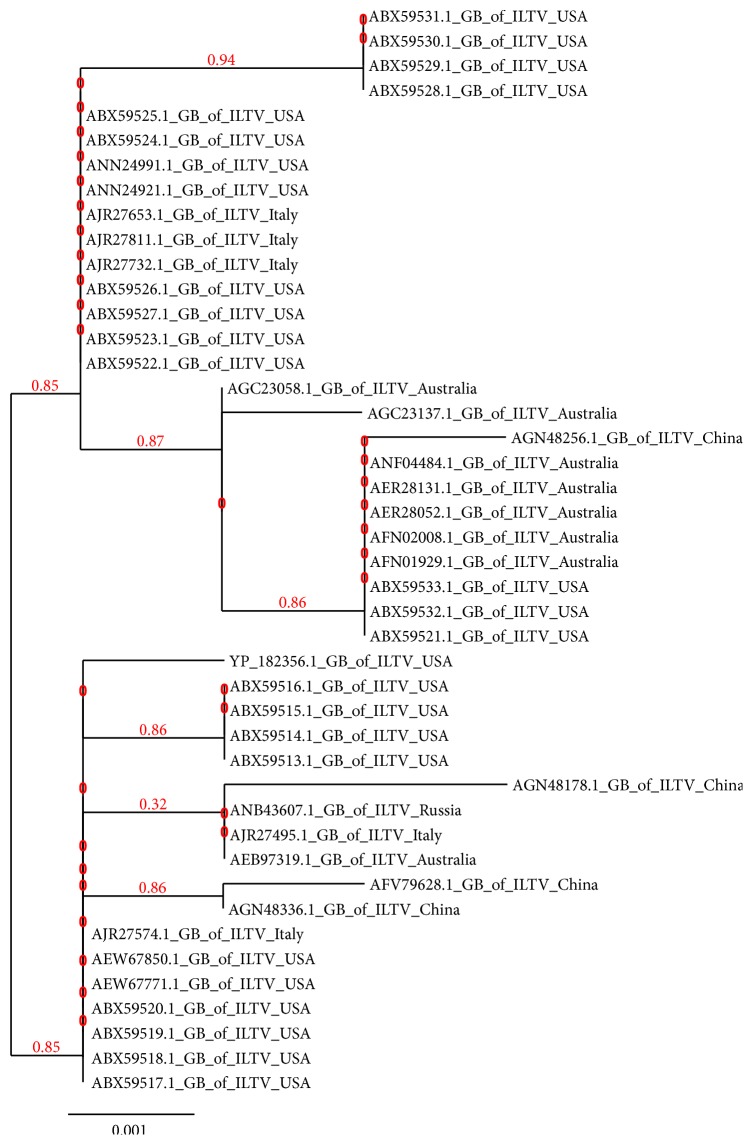
Evolutionary divergence analysis of enveloped glycoprotein B (GB) of different strains of ILTV.

Phylogenetic tree was created using (http://www.phylogeny.fr). The evolutionary divergence analysis of the enveloped glycoprotein B of the different strains of ILTV is presented in Figure 3.

### 3.3. Prediction of B Cell Epitopes

**Figure 4:**
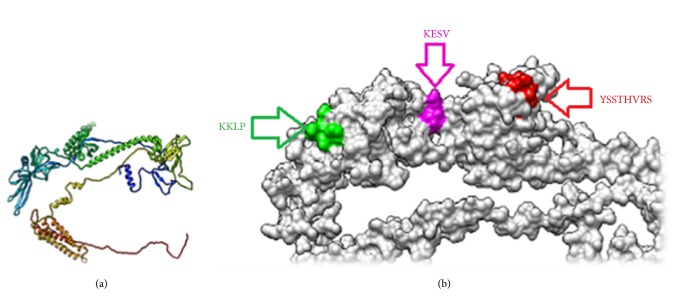
(a) The reference glycoprotein B of ILTV. (b) The position of proposed B cell epitopes in the 3D structure of reference glycoprotein B of ILTV.

Surface accessibility, hydrophilicity, flexibility, and antigenicity are important B cell antigenic indexes to design peptide vaccine. Investigation of ILTV glycoprotein B using different prediction methods of B cell at the Immune Epitope Database (IEDB) revealed varying threshold for different scales (see Figure 1). Thirty-one unique linear epitopes with 4 peptides or more in length were predicted using Bepipred Linear Epitope Prediction method depending on binding affinity to B lymphocytes. Analysis of these epitopes for surface accessibility and antigenicity proposed seventeen and thirteen peptides works as surface and antigenic epitopes, respectively (see Table 2). The predicted epitopes were found of high conservancy when tested in aligned sequences. Of these, only eight epitopes successfully covered all the antigenic indexes of B cell prediction tests. The best B cell predicted epitopes that overlap all B cell prediction methods were _190_*KKLP*_193_, _386_*YSSTHVRS*_393_, and _317_*KESV*_320_. The 3D structure of these predicted epitopes is shown in Figure 4.

### 3.4. Prediction of T Cell Epitopes

CD8+ and CD4+ T cells have principal role in stimulation of immune response as well as antigen mediated clonal expression of B cell [14]. Several technical problems challenged the design peptide vaccine against ILTV based on T cytotoxic and T helper epitopes prediction, most importantly, the lack of online bioinformatics database for chicken MHC alleles. For this reason human MHC class I alleles (HLA-A and HLA-B) were used in this study as an alternate alleles to investigate the interaction of epitopes with MHCI using epitope prediction software [43]. Studies have shown that the MHC genes in chickens are classified into MHCI associated genes (B-F) and MHCII (B-L) associated B-G genes [44]. The B-F alleles in chicken were found to be similar in stimulation of immune system to mammalian class I homologs especially in presenting the antigen of T-lymphocyte [45, 46]. MHC class I molecule in the chicken especially BF2^∗^2101 from the B21 haplotype is highly expressed, leading to strong genetic links with infectious pathogens. In addition, BF2^∗^2101 from the B21 haplotype has principal role in provoking resistance to Marek's disease caused by an oncogenic herpesvirus [43], to which ILTV belongs.

#### 3.4.1. Prediction of Epitopes Interacted with MHC Class I

**Figure 5:**
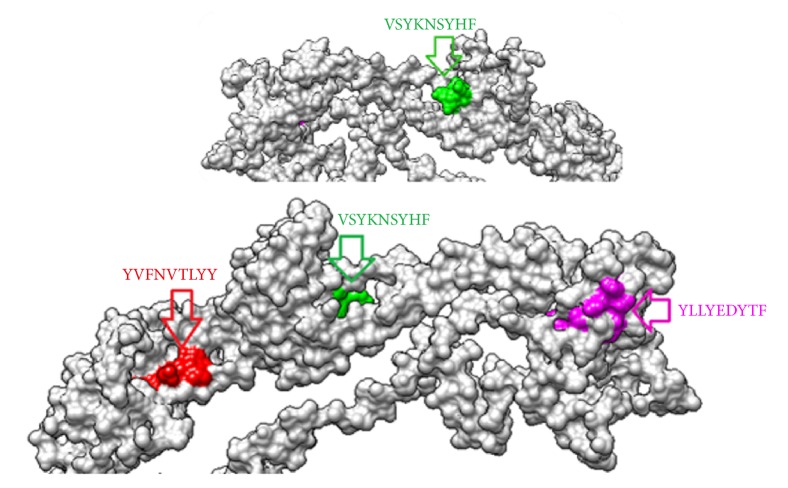
The 3D structure of reference glycoprotein B of ILTV and the position of proposed cytotoxic T cell epitopes suggested to interact with MHC-I virus illustrated by UCSF-Chimera visualization tool.

MHC-1 binding prediction tool using IEDB database predicted sixteen epitopes that interacted with the cytotoxic T cell as they strongly linked with multiple alleles. As shown in Table 3, MHCI results expected several CTL epitopes. The top epitope was _118_*YVFNVTLYY*_126_ which interacted and linked with 16 human alleles, followed by _335_*VSYKNSYHF*_343_ and _622_*YLLYEDYTF*_630_ as they linked with 9 human MHCI alleles. The 3D structure of the proposed epitopes is shown in Figure 5.

#### 3.4.2. Prediction of T Helper Cell Epitopes and Interaction with MHC Class II

**Figure 6:**
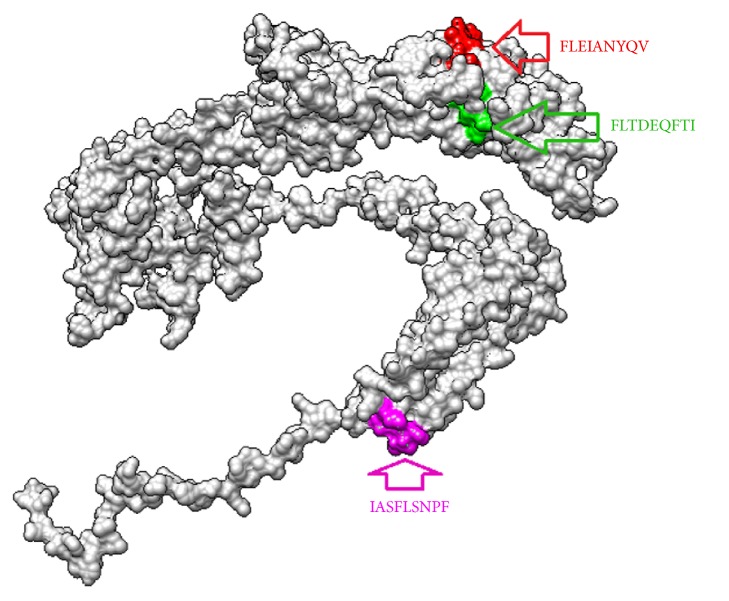
3D structure of reference glycoprotein B of ILTV and the position of proposed helper T cell epitopes suggested to interact with MHC-II virus illustrated by UCSF-Chimera visualization tool.

The reference strain of glycoprotein B was subjected to different analyzing methods using IEDB MHC-II binding prediction tool based on NN-align with half-maximal inhibitory concentration (IC50) ≤ 1000. The peptide (core) _301_*FLTDEQFTI*_309_ exhibited high affinity to MHCII alleles due to great binding with 76 MHC-II alleles followed by _277_*FLEIANYQV*_285_ and _743_*IASFLSNPF*_751_ as they linked with 67 human alleles (Table 4 and Figure 6).

### 3.5. Overlapping of T Cell Epitopes Residues in MHC Classes I and II

Twelve epitopes from top five proposed MHC class I were associated with at least to 15 alleles from MHC class II epitopes (see Table 5). It was observed that top proposed epitope from MHCII (*FLTDEQFTI*), which achieved the highest linkages with 76 alleles from MHC class II, was linked with 3 alleles only from MHCI. While the second proposed epitopes (*FLEIANYQV* and* IASFLSNPF*) that bound to 67 MHCII alleles were associated with 2 and 6 alleles from MHC1, respectively. However, of these top epitopes, only four of top MHC1 epitopes and two of best epitopes from MHCII were not linked to any alleles from MHCII and MHCI, respectively.

### 3.6. Molecular Docking of B-F Alleles and Predicted CTL Epitopes

**Figure 7:**
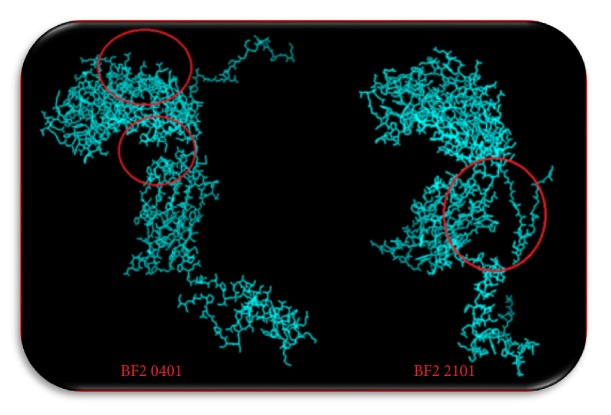
The 3D structure of BF_2_ alleles of chicken using Chimera visualization tool. Red circle indicated the binding site of epitopes.

**Figure 8:**
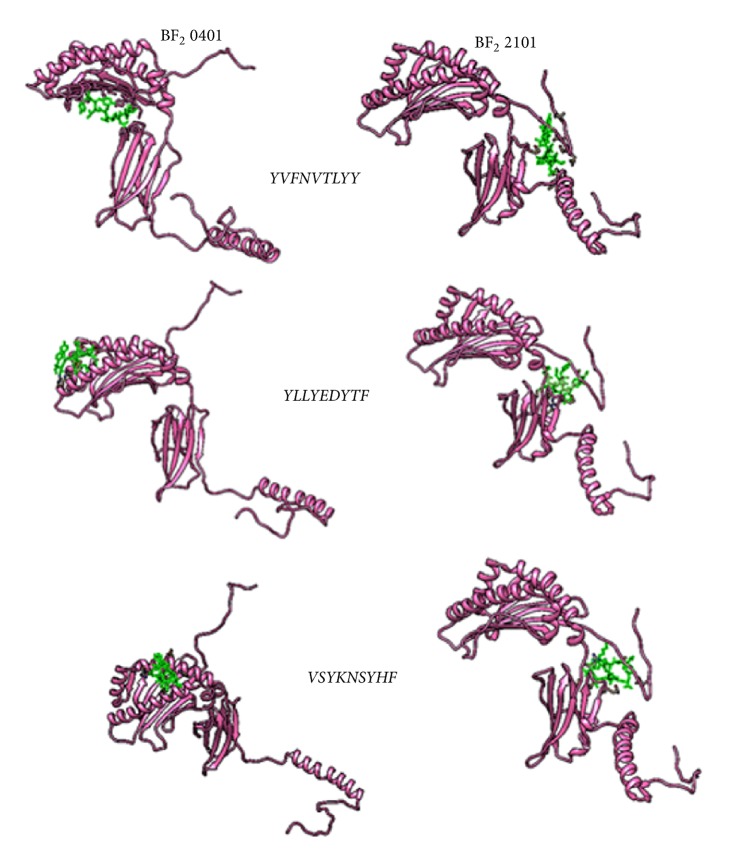
Visualization of PatchDock Molecular docking of MHC-I proposed epitopes and chicken BF2 alleles receptors using UCSF-Chimera visualization tool. Receptors (BF alleles) are represented by pink colour while CTL epitopes are represented by green one.

**Figure 9:**
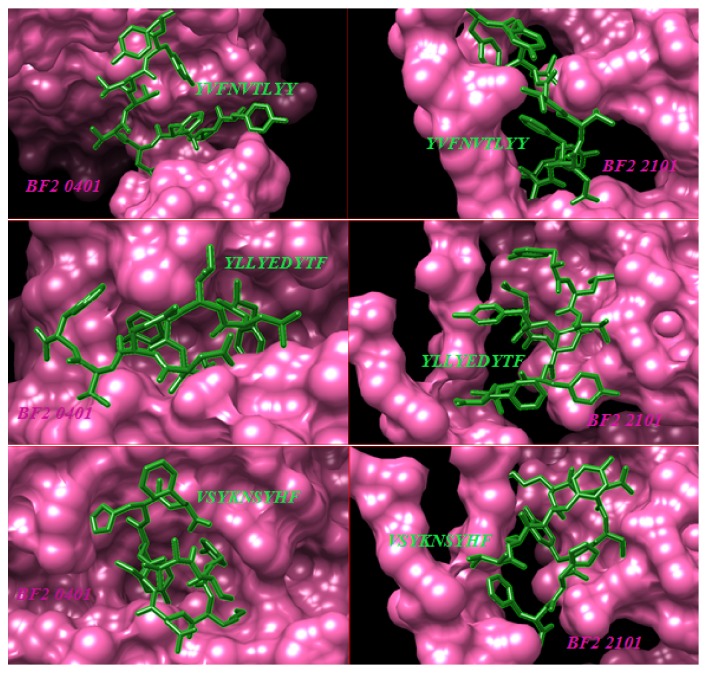
Visualization of PatchDock Molecular docking of MHCI proposed epitopes and chicken BF2 alleles receptors using UCSF-Chimera visualization tool. Receptors (BF alleles) are represented by rounded ribbon structure hot pink colour while CTL epitopes are represented by green one.

The top ranked CTL proposed epitopes were selected for molecular docking to predict and symbolize the image of real CTL epitopes interaction with chicken alleles. For this purpose, two types of chicken BF alleles (BF2*∗*2101; BF2*∗*0401) were selected. The docked epitopes (_301_*FLTDEQFTI*_309_, _277_*FLEIANYQV*_285_, and _743_*IASFLSNPF*_751_) using peptide-binding groove affinity were used to evaluate the ability of predicted epitopes to bind with chicken BF alleles/receptors to chicken alleles BF2 (BF2*∗*2101 and BF2*∗*0401). Results indicated that the docked epitopes achieved strong binding affinity to Chicken BF_2_ alleles based on global energy and attractive VDW in kcal/mol unit. The lowest binding energy (kcal/mol) was selected to predict probable CTL epitopes. Docked ligand epitopes (_118_*YVFNVTLYY*_126_, _622_*YLLYEDYTF*_630_, and _335_*VSYKNSYHF*_343_) with BF2 2101 alleles (receptor) showed higher binding affinity which expressed by the lower global energy (-91.78, -89.53, and -66.41, respectively). However, BF 2 0401 allele as a receptor produced less binding affinity with docked ligands (-45.65, -51.56, and -61.68, respectively). These results indicated that the binding affinity of ligands is higher with the receptor BF2*∗*2101 allele compared with the other allele (BF2 0401) which produced less binding affinity. In addition, the docked molecules showed different groove binding site for both BF alleles.  Figure 7 presents the 3D structure of chicken BF2 alleles and the proposed binding sites of docked epitopes. The binding energy scores in both BF2 alleles for the suggested epitopes using Patch Dock server for molecular docking are shown in Table 6. The visualization of the binding interactions between chicken BF2 receptor and MHCI epitopes in the structural level was performed using UCSF-Chimera visualization tool 1.8 (see Figures 8 and 9).

## 4. Conclusion

Smart computational techniques which provide tremendous predictive and analytical information facilitate the prediction of novel epitopes that may act as a powerful vaccine through immunoinformatic technology.

This is the first in silico study to design peptide vaccine against avian ILTV through humoral and cell mediated immune responses. The expected epitopes in this study could help in prevention of latent infection caused by the use of attenuated vaccines and developing more effective and trustable prophylactic and therapeutic vaccines than conventional methods.

In this study new epitopes were proposed as promising multiepitopes vaccine for ILTV. CTL epitopes were selected as vaccine candidates due to their high binding affinity with different alleles. The result should be supported by designing the peptide vaccine in the lab and through clinical trials.

## Figures and Tables

**Table 1:** Retrieved strains of ILTV with their date of collection, accession numbers, and geographical regions.

Accession No	country	Year	Accession No	country	Year
YP_182356.1*∗*	USA	2005	AEW67850.1	USA	2012
AFV79628.1	China	2011	AEW67771.1	USA	2011
ABX59525.1	＇＇USA	2007	AEB97319.1	Australia	2010
ABX59524.1	USA	2007	ABX59533.1	USA	2007
ANB43607.1	Russia	2000	ABX59532.1	USA	2007
ANF04484.1	Australia	2015	ABX59531.1	USA	2007
ANN24991.1	USA	2017	ABX59530.1	USA	2007
ANN24921.1	USA	2016	ABX59529.1	USA	2007
AJR27653.1	Italy	2007	ABX59528.1	USA	2007
AJR27811.1	Italy	1980	ABX59527.1	USA	2007
AJR27732.1	Italy	2011	ABX59526.1	USA	2007
AJR27574.1	Italy	2015	ABX59523.1	USA	2007
AJR27495.1	Italy	2015	ABX59522.1	USA	2007
AER28131.1	Australia	2011	ABX59521.1	USA	2007
AER28052.1	Australia	2011	ABX59520.1	USA	2007
AGN48336.1	China	2012	ABX59519.1	USA	2007
AGN48256.1	China	2012	ABX59518.1	USA	2007
AGN48178.1	China	2009	ABX59517.1	USA	2007
AGC23137.1	Australia	1970	ABX59516.1	USA	2007
AGC23058.1	Australia	1999	ABX59515.1	USA	2007
AFN02008.1	Australia	2011	ABX59514.1	USA	2007
AFN01929.1	Australia	2011	ABX59513.1	USA	2007

*∗*Refseq of ILTV envelope glycoprotein B.

**Table 2:** List of B cell epitopes predicted by different B cell scales.

No.	Peptide	Start	End	Length	Emini 1000	Antigenicity 1.027
1	QFTI	306	309	4	0.514	1.042
2	GQPVS	518	522	5	0.678	1.07
3	KLNPNS	506	511	6	1.715	0.968
4	NASEIE	635	640	6	0.888	0.951
5	LGEVGKA	718	724	7	0.303	1.032
6	DAMEEKESV	312	320	9	1.355	0.959
7	KESV*∗*	317	320	4	1.168	1.044
8	EVPEAVRVS	328	336	9	0.395	0.959
9	VIRGDRGDA	697	705	9	0.433	0.981
10	PQITNEYVTR	141	150	10	1.433	1.009
11	EYVTR*∗*	146	150	5	1.465	1.035
12	TFSSGKQPFN	351	360	10	0.957	0.977
13	RSGECSSKATY	163	173	11	0.837	1.01
14	YDNDEAEKKLP	183	193	11	4.592	0.964
15	KKLP*∗*	190	193	4	1.729	1.044
16	KDEQKARRQKA	834	844	11	11.486	0.946
17	EAIGSGAPKEPQI	58	70	13	0.374	0.997
18	HCHRHADSTNMTE	93	105	13	0.786	0.986
19	CSSPTGASVARLAQ	77	90	14	0.103	1.072
20	YSSTHVRSGDIEYYL	386	400	15	0.537	1.052
21	YSSTHVRS*∗*	386	393	8	1.152	1.058
22	NFTKRHQTLGYRTSTS	211	226	16	2.246	0.976
23	RHQTLGY*∗*	215	221	7	1.232	1.027
24	SSSPESQFSANSTENH	569	584	16	1.851	0.973
25	VYTREELRDTGTLNYD	663	678	16	1.703	0.985
26	VYTREEL*∗*	663	669	7	1.176	1.04
27	VRDLETGQIRPPKKRNFL	285	302	18	1.191	1.001
28	QIRPP*∗*	292	296	5	1.463	1.034
29	FGMATGDTVEISPFYTKNTTGPRRHSV	245	271	27	0.185	0.991
30	EEAQRQNHLPRGRERRQAAGRRTASLQSGPQGDRITTHSS	423	462	40	8.232	0.969
31	QNHLP*∗*	428	432	5	1.243	1.042

*∗*Shortened peptide that has high score in both Emini and Kolaskar.

**Table 3:** Position of most promising epitopes in the glycoprotein B of ILTV that bind with high affinity with the human MHC class I alleles.

Peptide	Start	End	Allele	ic50
MLICVCVAI	16	24	HLA-A*∗*02:01	19.5
			HLA-A*∗*02:06	27.85
			HLA-A*∗*32:01	181.4
			HLA-A*∗*68:02	50.12
			HLA-B*∗*15:01	209.67

YVFNVTLYY*∗*	118	126	HLA-A*∗*01:01	52.47
			HLA-A*∗*03:01	31.62
			HLA-A*∗*11:01	8.01
			HLA-A*∗*25:01	245.85
			HLA-A*∗*26:01	7.03
			HLA-A*∗*29:02	2.26
			HLA-A*∗*30:02	29.28
			HLA-A*∗*68:01	10.69
			HLA-B*∗*15:01	41.17
			HLA-B*∗*35:01	8.03
			HLA-B*∗*46:01	109.43
			HLA-B*∗*58:01	103.63

LYYKHITTV	124	132	HLA-A*∗*23:01	176.88
			HLA-A*∗*24:02	255.62
			HLA-C*∗*06:02	228.43
			HLA-C*∗*07:01	281.81
			HLA-C*∗*12:03	40.39
			HLA-C*∗*14:02	20.88

TTVTTWALF	130	138	HLA-A*∗*23:01	186.44
			HLA-A*∗*24:02	291.18
			HLA-A*∗*26:01	43.17
			HLA-A*∗*29:02	140.22
			HLA-B*∗*57:01	135.77
			HLA-B*∗*58:01	126.76

MATGDTVEI	247	255	HLA-A*∗*02:06	298.31
			HLA-A*∗*68:02	27.18
			HLA-B*∗*35:01	70.95
			HLA-B*∗*53:01	91.98
			HLA-C*∗*03:03	22.67
			HLA-C*∗*12:03	107.28

DTVEISPFY	251	259	HLA-A*∗*25:01	219.57
			HLA-A*∗*26:01	2.97
			HLA-A*∗*29:02	28.64
			HLA-A*∗*68:01	41.47
			HLA-B*∗*35:01	269.44

YRFLEIANY	275	283	HLA-B*∗*27:05	183.15
			HLA-C*∗*06:02	157.29
			HLA-C*∗*07:01	169.97
			HLA-C*∗*07:02	281.69
			HLA-C*∗*12:03	252.46
			HLA-C*∗*14:02	290.98

VSYKNSYHF*∗*	335	343	HLA-A*∗*23:01	26.91
			HLA-A*∗*24:02	283.17
			HLA-A*∗*32:01	256.64
			HLA-B*∗*15:01	45.93
			HLA-B*∗*57:01	69.13
			HLA-B*∗*58:01	18.43
			HLA-C*∗*03:03	254.73
			HLA-C*∗*12:03	111.22
			HLA-C*∗*15:02	261.59

YKNSYHFSL	337	345	HLA-B*∗*08:01	212.76
			HLA-B*∗*39:01	7.84
			HLA-C*∗*03:03	17.48
			HLA-C*∗*07:02	41.18
			HLA-C*∗*14:02	249.85

HVRSGDIEY	390	398	HLA-A*∗*29:02	250.74
			HLA-A*∗*30:01	43.1
			HLA-B*∗*15:01	199.81
			HLA-B*∗*15:02	295.16
			HLA-B*∗*35:01	9.19

MSHGLAEMY	413	421	HLA-A*∗*29:02	33.03
			HLA-A*∗*30:02	95.04
			HLA-B*∗*15:01	263.61
			HLA-B*∗*35:01	49.68
			HLA-B*∗*57:01	132.85
			HLA-B*∗*58:01	228.7

FAYDKIQAH	470	478	HLA-B*∗*35:01	56.25
			HLA-B*∗*46:01	189.03
			HLA-C*∗*03:03	5.42
			HLA-C*∗*12:03	9.1
			HLA-C*∗*14:02	231.14

YLLYEDYTF*∗*	622	630	HLA-A*∗*02:01	215.94
			HLA-A*∗*02:06	77.51
			HLA-A*∗*23:01	31.7
			HLA-A*∗*24:02	237.46
			HLA-A*∗*29:02	60.11
			HLA-A*∗*32:01	132.77
			HLA-B*∗*15:01	163.87
			HLA-B*∗*15:02	48.14
			HLA-B*∗*35:01	62.28

VVMTAAAAV	728	736	HLA-A*∗*02:01	80.99
			HLA-A*∗*02:06	10.14
			HLA-A*∗*68:02	42.19
			HLA-C*∗*14:02	60.62
			HLA-C*∗*15:02	231.69

IASFLSNPF	743	751	HLA-A*∗*32:01	81.65
			HLA-B*∗*15:01	94.19
			HLA-B*∗*35:01	11.95
			HLA-B*∗*58:01	219.61
			HLA-C*∗*03:03	83.79
			HLA-C*∗*12:03	189.81

FLSNPFAAL	746	754	HLA-A*∗*02:01	16.28
			HLA-A*∗*02:06	8.88
			HLA-A*∗*68:02	172.75
			HLA-B*∗*15:01	128.57
			HLA-B*∗*15:02	136.86
			HLA-C*∗*03:03	3.25
			HLA-C*∗*12:03	140.95
			HLA-C*∗*14:02	85.38

KSNPVQVLF	778	786	HLA-A*∗*32:01	76.58
			HLA-B*∗*15:01	283.12
			HLA-B*∗*57:01	19.71
			HLA-B*∗*58:01	2.62
			HLA-C*∗*15:02	155.84

*∗*Proposed peptides.

**Table 4:** List of best six epitopes that bind with high affinity with the human MHC class II alleles.

Core Sequence	Peptide Sequence	Start	End	Allele	IC50
LLRSTVSKA	LPLVPSLLRSTVSKA	192	206	HLA-DQA1*∗*01:02/DQB1*∗*06:02	824.7
				HLA-DRB1*∗*01:01	99.4
				HLA-DRB1*∗*03:01	421.6
				HLA-DRB1*∗*04:01	300.9
				HLA-DRB1*∗*04:05	768.9
				HLA-DRB1*∗*07:01	155.6
				HLA-DRB1*∗*08:02	143.9
				HLA-DRB1*∗*09:01	783.8
				HLA-DRB1*∗*15:01	888.9
	PLVPSLLRSTVSKAF	193	207	HLA-DPA1*∗*02:01/DPB1*∗*01:01	436
				HLA-DQA1*∗*01:02/DQB1*∗*06:02	538.2
				HLA-DQA1*∗*05:01/DQB1*∗*03:01	413.8
				HLA-DRB1*∗*01:01	42.1
				HLA-DRB1*∗*03:01	123.5
				HLA-DRB1*∗*04:01	185.5
				HLA-DRB1*∗*04:04	166.7
				HLA-DRB1*∗*04:05	478
				HLA-DRB1*∗*08:02	103
				HLA-DRB1*∗*09:01	394.9
				HLA-DRB1*∗*13:02	774.7
				HLA-DRB1*∗*15:01	978
	LVPSLLRSTVSKAFH	194	208	HLA-DPA1*∗*02:01/DPB1*∗*01:01	464.3
				HLA-DQA1*∗*01:02/DQB1*∗*06:02	457.4
				HLA-DQA1*∗*05:01/DQB1*∗*03:01	378.5
				HLA-DRB1*∗*01:01	26.9
				HLA-DRB1*∗*03:01	57.1
				HLA-DRB1*∗*04:01	147.6
				HLA-DRB1*∗*04:05	360
				HLA-DRB1*∗*08:02	71.5
				HLA-DRB1*∗*09:01	336.5
				HLA-DRB1*∗*11:01	148.8
				HLA-DRB1*∗*13:02	652.3
				HLA-DRB1*∗*15:01	824.6
	VPSLLRSTVSKAFHT	195	209	HLA-DPA1*∗*02:01/DPB1*∗*01:01	682.2
				HLA-DQA1*∗*01:02/DQB1*∗*06:02	397.2
				HLA-DRB1*∗*01:01	18
				HLA-DRB1*∗*03:01	32.8
				HLA-DRB1*∗*04:01	108.8
				HLA-DRB1*∗*04:05	303.1
				HLA-DRB1*∗*08:02	52
				HLA-DRB1*∗*09:01	219.9
				HLA-DRB1*∗*13:02	568.1
				HLA-DRB1*∗*15:01	745
	PSLLRSTVSKAFHTT	196	210	HLA-DPA1*∗*02:01/DPB1*∗*01:01	871.8
				HLA-DQA1*∗*01:02/DQB1*∗*06:02	506.4
				HLA-DRB1*∗*01:01	26.3
				HLA-DRB1*∗*03:01	49.1
				HLA-DRB1*∗*04:01	126.7
				HLA-DRB1*∗*04:04	178.1
				HLA-DRB1*∗*04:05	369.2
				HLA-DRB1*∗*08:02	61.5
				HLA-DRB1*∗*15:01	809.3
	SLLRSTVSKAFHTTN	197	211	HLA-DQA1*∗*01:02/DQB1*∗*06:02	609.2
				HLA-DRB1*∗*01:01	36.6
				HLA-DRB1*∗*03:01	89.2
				HLA-DRB1*∗*04:01	130.9
				HLA-DRB1*∗*04:05	467.4
				HLA-DRB1*∗*08:02	75.6
	LLRSTVSKAFHTTNF	198	212	HLA-DQA1*∗*01:02/DQB1*∗*06:02	836.1
				HLA-DRB1*∗*03:01	218.7
				HLA-DRB1*∗*04:01	184.1
				HLA-DRB1*∗*04:04	384.5
				HLA-DRB1*∗*04:05	643.2
				HLA-DRB1*∗*08:02	106

FLEIANYQV	VYRDYRFLEIANYQV	271	285	HLA-DRB1*∗*07:01	9.1
				HLA-DRB1*∗*08:02	752
				HLA-DRB1*∗*13:02	288.3
				HLA-DRB5*∗*01:01	67.4
	YRDYRFLEIANYQVR	272	286	HLA-DRB1*∗*07:01	10.9
				HLA-DRB1*∗*08:02	565
				HLA-DRB1*∗*13:02	237.5
				HLA-DRB5*∗*01:01	29.8
	RDYRFLEIANYQVRD	273	287	HLA-DPA1*∗*01/DPB1*∗*04:01	284.1
				HLA-DPA1*∗*01:03/DPB1*∗*02:01	90.8
				HLA-DRB1*∗*01:01	8.9
				HLA-DRB1*∗*04:01	41.8
				HLA-DRB1*∗*07:01	15.6
				HLA-DRB1*∗*08:02	585.2
				HLA-DRB1*∗*11:01	54.1
				HLA-DRB1*∗*13:02	216.2
				HLA-DRB3*∗*01:01	875
				HLA-DRB5*∗*01:01	27.2
				HLA-DPA1*∗*02:01/DPB1*∗*05:01	218.5
				HLA-DQA1*∗*01:01/DQB1*∗*05:01	637.4
				HLA-DQA1*∗*01:02/DQB1*∗*06:02	331.2
				HLA-DRB1*∗*04:04	81.1
				HLA-DRB1*∗*04:05	32.1
				HLA-DRB1*∗*09:01	273.4
	DYRFLEIANYQVRDL	274	288	HLA-DPA1*∗*01/DPB1*∗*04:01	475
				HLA-DPA1*∗*01:03/DPB1*∗*02:01	72.5
				HLA-DPA1*∗*02:01/DPB1*∗*01:01	42.9
				HLA-DPA1*∗*02:01/DPB1*∗*05:01	294.4
				HLA-DPA1*∗*03:01/DPB1*∗*04:02	462.5
				HLA-DRB1*∗*01:01	8.3
				HLA-DRB1*∗*04:01	58.2
				HLA-DRB1*∗*04:05	39.5
				HLA-DRB1*∗*07:01	15.9
				HLA-DRB1*∗*08:02	704.3
				HLA-DRB1*∗*11:01	43.5
				HLA-DRB1*∗*13:02	190.3
				HLA-DRB3*∗*01:01	979.2
				HLA-DRB5*∗*01:01	18.3
	RFLEIANYQVRDLET	276	290	HLA-DPA1*∗*01/DPB1*∗*04:01	594
				HLA-DPA1*∗*01:03/DPB1*∗*02:01	113.7
				HLA-DPA1*∗*02:01/DPB1*∗*01:01	66
				HLA-DPA1*∗*02:01/DPB1*∗*05:01	726.4
				HLA-DRB1*∗*01:01	15.5
				HLA-DRB1*∗*04:01	261.2
				HLA-DRB1*∗*04:04	174.2
				HLA-DRB1*∗*04:05	174.3
				HLA-DRB1*∗*07:01	31
				HLA-DRB1*∗*09:01	931.6
				HLA-DRB1*∗*11:01	140.6
				HLA-DRB1*∗*13:02	486.4
				HLA-DRB5*∗*01:01	39
	FLEIANYQVRDLETG	277	291	HLA-DPA1*∗*01:03/DPB1*∗*02:01	336.8
				HLA-DPA1*∗*02:01/DPB1*∗*01:01	146.9
				HLA-DRB1*∗*01:01	28.9
				HLA-DRB1*∗*04:04	273.3
				HLA-DRB1*∗*04:05	325.2
				HLA-DRB1*∗*07:01	57.2
				HLA-DRB1*∗*11:01	340.9
				HLA-DRB1*∗*13:02	852.9
				HLA-DRB5*∗*01:01	101.7

FLTDEQFTI	PPKKRNFLTDEQFTI	295	309	HLA-DPA1*∗*01/DPB1*∗*04:01	246.4
				HLA-DPA1*∗*01:03/DPB1*∗*02:01	35.5
				HLA-DPA1*∗*02:01/DPB1*∗*01:01	93.6
				HLA-DPA1*∗*02:01/DPB1*∗*05:01	541
				HLA-DPA1*∗*03:01/DPB1*∗*04:02	506.6
				HLA-DRB1*∗*03:01	76.1
				HLA-DRB1*∗*04:01	198.7
				HLA-DRB1*∗*04:05	274.9
				HLA-DRB1*∗*07:01	98.6
				HLA-DRB3*∗*01:01	4.9
				HLA-DRB5*∗*01:01	948.8
	PKKRNFLTDEQFTIG	296	310	HLA-DPA1*∗*01/DPB1*∗*04:01	172.7
				HLA-DPA1*∗*01:03/DPB1*∗*02:01	24.5
				HLA-DPA1*∗*02:01/DPB1*∗*01:01	82.3
				HLA-DPA1*∗*02:01/DPB1*∗*05:01	475.5
				HLA-DPA1*∗*03:01/DPB1*∗*04:02	341.5
				HLA-DRB1*∗*01:01	850.7
				HLA-DRB1*∗*03:01	59.9
				HLA-DRB1*∗*04:01	136.1
				HLA-DRB1*∗*04:04	869.9
				HLA-DRB1*∗*04:05	325
				HLA-DRB1*∗*07:01	162
				HLA-DRB3*∗*01:01	4.8
				HLA-DRB5*∗*01:01	769
	KKRNFLTDEQFTIGW	297	311	HLA-DPA1*∗*01/DPB1*∗*04:01	123
				HLA-DPA1*∗*01:03/DPB1*∗*02:01	20.1
				HLA-DPA1*∗*02:01/DPB1*∗*01:01	66.4
				HLA-DPA1*∗*02:01/DPB1*∗*05:01	394.6
				HLA-DPA1*∗*03:01/DPB1*∗*04:02	199
				HLA-DRB1*∗*01:01	342.9
				HLA-DRB1*∗*03:01	37.7
				HLA-DRB1*∗*04:01	103.7
				HLA-DRB1*∗*04:04	805.9
				HLA-DRB1*∗*04:05	330.3
				HLA-DRB1*∗*07:01	203.9
				HLA-DRB3*∗*01:01	4.6
				HLA-DRB5*∗*01:01	527
	KRNFLTDEQFTIGWD	298	312	HLA-DPA1*∗*01/DPB1*∗*04:01	110.8
				HLA-DPA1*∗*01:03/DPB1*∗*02:01	21.6
				HLA-DPA1*∗*02:01/DPB1*∗*01:01	65
				HLA-DPA1*∗*02:01/DPB1*∗*05:01	405.2
				HLA-DPA1*∗*03:01/DPB1*∗*04:02	148.2
				HLA-DRB1*∗*01:01	354.3
				HLA-DRB1*∗*03:01	31.7
				HLA-DRB1*∗*04:01	98.2
				HLA-DRB1*∗*04:05	398.7
				HLA-DRB1*∗*07:01	325.7
				HLA-DRB3*∗*01:01	4.4
				HLA-DRB5*∗*01:01	505.9
	RNFLTDEQFTIGWDA	299	313	HLA-DPA1*∗*01/DPB1*∗*04:01	115.3
				HLA-DPA1*∗*01:03/DPB1*∗*02:01	26.7
				HLA-DPA1*∗*02:01/DPB1*∗*01:01	88.5
				HLA-DPA1*∗*02:01/DPB1*∗*05:01	532.9
				HLA-DPA1*∗*03:01/DPB1*∗*04:02	182.5
				HLA-DRB1*∗*01:01	687.7
				HLA-DRB1*∗*03:01	64.7
				HLA-DRB1*∗*04:01	178.3
				HLA-DRB1*∗*04:05	700.1
				HLA-DRB3*∗*01:01	5.9
				HLA-DRB5*∗*01:01	948.3
	NFLTDEQFTIGWDAM	300	314	HLA-DPA1*∗*01/DPB1*∗*04:01	244
				HLA-DPA1*∗*01:03/DPB1*∗*02:01	44.6
				HLA-DPA1*∗*02:01/DPB1*∗*01:01	173.3
				HLA-DPA1*∗*03:01/DPB1*∗*04:02	250.2
				HLA-DQA1*∗*05:01/DQB1*∗*02:01	475.1
				HLA-DRB1*∗*03:01	164.3
				HLA-DRB1*∗*04:01	278.3
				HLA-DRB3*∗*01:01	9
	FLTDEQFTIGWDAME	301	315	HLA-DPA1*∗*01/DPB1*∗*04:01	640
				HLA-DPA1*∗*01:03/DPB1*∗*02:01	154.8
				HLA-DPA1*∗*02:01/DPB1*∗*01:01	395.6
				HLA-DPA1*∗*03:01/DPB1*∗*04:02	437.8
				HLA-DQA1*∗*05:01/DQB1*∗*02:01	459.5
				HLA-DRB1*∗*03:01	433.9
				HLA-DRB1*∗*04:01	271.4
				HLA-DRB3*∗*01:01	15.5

LLGDIVAVS	QPVSARLLGDIVAVS	519	533	HLA-DPA1*∗*02:01/DPB1*∗*01:01	922.5
				HLA-DPA1*∗*03:01/DPB1*∗*04:02	822.3
				HLA-DQA1*∗*01:02/DQB1*∗*06:02	726
				HLA-DQA1*∗*05:01/DQB1*∗*03:01	42.2
				HLA-DRB1*∗*04:01	494.1
				HLA-DRB1*∗*08:02	914.5
				HLA-DRB3*∗*01:01	211.5
	PVSARLLGDIVAVSK	520	534	HLA-DPA1*∗*02:01/DPB1*∗*01:01	752.7
				HLA-DPA1*∗*03:01/DPB1*∗*04:02	853.4
				HLA-DQA1*∗*01:02/DQB1*∗*06:02	715.4
				HLA-DQA1*∗*05:01/DQB1*∗*03:01	34
				HLA-DRB1*∗*03:01	380.3
				HLA-DRB1*∗*04:01	298
				HLA-DRB1*∗*04:05	458.8
				HLA-DRB1*∗*08:02	571.8
				HLA-DRB3*∗*01:01	196.5
	VSARLLGDIVAVSKC	521	535	HLA-DPA1*∗*02:01/DPB1*∗*01:01	723.1
				HLA-DPA1*∗*03:01/DPB1*∗*04:02	733.4
				HLA-DQA1*∗*01:02/DQB1*∗*06:02	502.8
				HLA-DQA1*∗*05:01/DQB1*∗*03:01	29.9
				HLA-DRB1*∗*03:01	214.3
				HLA-DRB1*∗*04:01	237
				HLA-DRB1*∗*04:05	429.4
				HLA-DRB1*∗*08:02	541.8
				HLA-DRB3*∗*01:01	172.3
	SARLLGDIVAVSKCI	522	536	HLA-DPA1*∗*02:01/DPB1*∗*01:01	677.6
				HLA-DPA1*∗*03:01/DPB1*∗*04:02	650.5
				HLA-DQA1*∗*01:02/DQB1*∗*06:02	555.2
				HLA-DQA1*∗*05:01/DQB1*∗*03:01	26.4
				HLA-DRB1*∗*01:01	175.3
				HLA-DRB1*∗*03:01	124.9
				HLA-DRB1*∗*04:01	187.9
				HLA-DRB1*∗*04:05	396.3
				HLA-DRB1*∗*08:02	396.7
				HLA-DRB3*∗*01:01	143.4
	ARLLGDIVAVSKCIE	523	537	HLA-DPA1*∗*02:01/DPB1*∗*01:01	791.2
				HLA-DPA1*∗*03:01/DPB1*∗*04:02	787.4
				HLA-DQA1*∗*01:02/DQB1*∗*06:02	590.2
				HLA-DQA1*∗*05:01/DQB1*∗*03:01	31.5
				HLA-DRB1*∗*01:01	270.9
				HLA-DRB1*∗*03:01	199.3
				HLA-DRB1*∗*04:01	255.3
				HLA-DRB1*∗*04:05	643.6
				HLA-DRB1*∗*08:02	360.5
				HLA-DRB3*∗*01:01	379.2
	RLLGDIVAVSKCIEI	524	538	HLA-DPA1*∗*02:01/DPB1*∗*01:01	807.2
				HLA-DPA1*∗*03:01/DPB1*∗*04:02	911.1
				HLA-DQA1*∗*01:02/DQB1*∗*06:02	621.6
				HLA-DQA1*∗*05:01/DQB1*∗*03:01	35.4
				HLA-DRB1*∗*03:01	347.3
				HLA-DRB1*∗*04:01	375.6
				HLA-DRB1*∗*04:05	977.9
				HLA-DRB1*∗*08:02	414.9
				HLA-DRB3*∗*01:01	974.2
	LLGDIVAVSKCIEIP	525	539	HLA-DPA1*∗*02:01/DPB1*∗*01:01	941
				HLA-DQA1*∗*01:02/DQB1*∗*06:02	748.6
				HLA-DQA1*∗*05:01/DQB1*∗*03:01	37.1
				HLA-DRB1*∗*03:01	688
				HLA-DRB1*∗*04:01	848.5
				HLA-DRB1*∗*08:02	521

IASFLSNPF	ISTVSGIASFLSNPF	737	751	HLA-DPA1*∗*02:01/DPB1*∗*01:01	264.9
				HLA-DQA1*∗*05:01/DQB1*∗*02:01	384.2
				HLA-DRB1*∗*04:01	53.5
				HLA-DRB1*∗*04:04	92.5
				HLA-DRB1*∗*04:05	16.2
				HLA-DRB1*∗*07:01	25
				HLA-DRB1*∗*09:01	104.9
				HLA-DRB1*∗*15:01	24.4
				HLA-DRB5*∗*01:01	103.5
	STVSGIASFLSNPFA	738	752	HLA-DPA1*∗*01/DPB1*∗*04:01	559.3
				HLA-DPA1*∗*01:03/DPB1*∗*02:01	320.6
				HLA-DPA1*∗*02:01/DPB1*∗*01:01	240.5
				HLA-DQA1*∗*01:01/DQB1*∗*05:01	976.4
				HLA-DQA1*∗*05:01/DQB1*∗*02:01	474.6
				HLA-DRB1*∗*01:01	101.3
				HLA-DRB1*∗*04:01	41.8
				HLA-DRB1*∗*04:05	15.5
				HLA-DRB1*∗*07:01	31.3
				HLA-DRB1*∗*08:02	589.8
				HLA-DRB1*∗*09:01	74.3
				HLA-DRB1*∗*15:01	18.9
				HLA-DRB5*∗*01:01	77.6
	TVSGIASFLSNPFAA	739	753	HLA-DPA1*∗*01:03/DPB1*∗*02:01	179.9
				HLA-DPA1*∗*02:01/DPB1*∗*01:01	219.5
				HLA-DQA1*∗*01:01/DQB1*∗*05:01	915.8
				HLA-DQA1*∗*05:01/DQB1*∗*02:01	611.8
				HLA-DRB1*∗*01:01	40.6
				HLA-DRB1*∗*04:01	27.7
				HLA-DRB1*∗*04:05	15.5
				HLA-DRB1*∗*07:01	44
				HLA-DRB1*∗*08:02	538.8
				HLA-DRB1*∗*09:01	72.2
				HLA-DRB1*∗*15:01	15.9
				HLA-DRB5*∗*01:01	57.1
	VSGIASFLSNPFAAL	740	754	HLA-DQA1*∗*01:01/DQB1*∗*05:01	710
				HLA-DQA1*∗*05:01/DQB1*∗*02:01	755.5
				HLA-DRB1*∗*01:01	14.2
				HLA-DRB1*∗*03:01	427.9
				HLA-DRB1*∗*04:01	22
				HLA-DRB1*∗*04:05	14.6
				HLA-DRB1*∗*07:01	43.3
				HLA-DRB1*∗*08:02	486.4
				HLA-DRB1*∗*09:01	52.2
				HLA-DRB1*∗*11:01	377.7
				HLA-DRB1*∗*15:01	12.8
				HLA-DRB5*∗*01:01	29.4
	SGIASFLSNPFAALG	741	755	HLA-DQA1*∗*01:01/DQB1*∗*05:01	936.1
				HLA-DRB1*∗*03:01	629.7
				HLA-DRB1*∗*04:01	27.6
				HLA-DRB1*∗*04:05	18
				HLA-DRB1*∗*07:01	67.9
				HLA-DRB1*∗*08:02	582.5
				HLA-DRB1*∗*09:01	56.2
				HLA-DRB1*∗*15:01	14.6
				HLA-DRB5*∗*01:01	50.2
	GIASFLSNPFAALGI	742	756	HLA-DRB1*∗*01:01	27.1
				HLA-DRB1*∗*04:01	38.1
				HLA-DRB1*∗*04:05	27.3
				HLA-DRB1*∗*08:02	594.2
				HLA-DRB1*∗*09:01	58.6
				HLA-DRB1*∗*15:01	17.4
				HLA-DRB5*∗*01:01	73.5
	IASFLSNPFAALGIG	743	757	HLA-DRB1*∗*04:01	67
				HLA-DRB1*∗*04:05	41.9
				HLA-DRB1*∗*15:01	28.6
				HLA-DRB4*∗*01:01	735.5
				HLA-DRB5*∗*01:01	119.8

LLGDIVAVS	QPVSARLLGDIVAVS	519	533	HLA-DPA1*∗*02:01/DPB1*∗*01:01	922.5
				HLA-DPA1*∗*03:01/DPB1*∗*04:02	822.3
				HLA-DQA1*∗*01:02/DQB1*∗*06:02	726
				HLA-DQA1*∗*05:01/DQB1*∗*03:01	42.2
				HLA-DRB1*∗*04:01	494.1
				HLA-DRB1*∗*08:02	914.5
				HLA-DRB3*∗*01:01	211.5
	PVSARLLGDIVAVSK	520	534	HLA-DPA1*∗*02:01/DPB1*∗*01:01	752.7
				HLA-DPA1*∗*03:01/DPB1*∗*04:02	853.4
				HLA-DQA1*∗*01:02/DQB1*∗*06:02	715.4
				HLA-DQA1*∗*05:01/DQB1*∗*03:01	34
				HLA-DRB1*∗*03:01	380.3
				HLA-DRB1*∗*04:01	298
				HLA-DRB1*∗*04:05	458.8
				HLA-DRB1*∗*08:02	571.8
				HLA-DRB3*∗*01:01	196.5
	VSARLLGDIVAVSKC	521	535	HLA-DPA1*∗*02:01/DPB1*∗*01:01	723.1
				HLA-DPA1*∗*03:01/DPB1*∗*04:02	733.4
				HLA-DQA1*∗*01:02/DQB1*∗*06:02	502.8
				HLA-DQA1*∗*05:01/DQB1*∗*03:01	29.9
				HLA-DRB1*∗*03:01	214.3
				HLA-DRB1*∗*04:01	237
				HLA-DRB1*∗*04:05	429.4
				HLA-DRB1*∗*08:02	541.8
				HLA-DRB3*∗*01:01	172.3
	SARLLGDIVAVSKCI	522	536	HLA-DPA1*∗*02:01/DPB1*∗*01:01	677.6
				HLA-DPA1*∗*03:01/DPB1*∗*04:02	650.5
				HLA-DQA1*∗*01:02/DQB1*∗*06:02	555.2
				HLA-DQA1*∗*05:01/DQB1*∗*03:01	26.4
				HLA-DRB1*∗*01:01	175.3
				HLA-DRB1*∗*03:01	124.9
				HLA-DRB1*∗*04:01	187.9
				HLA-DRB1*∗*04:05	396.3
				HLA-DRB1*∗*08:02	396.7
				HLA-DRB3*∗*01:01	143.4
	ARLLGDIVAVSKCIE	523	537	HLA-DPA1*∗*02:01/DPB1*∗*01:01	791.2
				HLA-DPA1*∗*03:01/DPB1*∗*04:02	787.4
				HLA-DQA1*∗*01:02/DQB1*∗*06:02	590.2
				HLA-DQA1*∗*05:01/DQB1*∗*03:01	31.5
				HLA-DRB1*∗*01:01	270.9
				HLA-DRB1*∗*03:01	199.3
				HLA-DRB1*∗*04:01	255.3
				HLA-DRB1*∗*04:05	643.6
				HLA-DRB1*∗*08:02	360.5
				HLA-DRB3*∗*01:01	379.2
	RLLGDIVAVSKCIEI	524	538	HLA-DPA1*∗*02:01/DPB1*∗*01:01	807.2
				HLA-DPA1*∗*03:01/DPB1*∗*04:02	911.1
				HLA-DQA1*∗*01:02/DQB1*∗*06:02	621.6
				HLA-DQA1*∗*05:01/DQB1*∗*03:01	35.4
				HLA-DRB1*∗*03:01	347.3
				HLA-DRB1*∗*04:01	375.6
				HLA-DRB1*∗*04:05	977.9
				HLA-DRB1*∗*08:02	414.9
				HLA-DRB3*∗*01:01	974.2
	LLGDIVAVSKCIEIP	525	539	HLA-DPA1*∗*02:01/DPB1*∗*01:01	941
				HLA-DQA1*∗*01:02/DQB1*∗*06:02	748.6
				HLA-DQA1*∗*05:01/DQB1*∗*03:01	37.1
				HLA-DRB1*∗*03:01	688
				HLA-DRB1*∗*04:01	848.5
				HLA-DRB1*∗*08:02	521

*∗*Inhibitory concentration needed for binding MHC II to the IEDB NN-align method; the lower value is better.

**Table 5:** Comparison between the numbers of alleles linked with top proposed epitopes in MHCI and MHCII.

Peptide	MHCI	MHCII
*MLICVCVAI*	5	0
*YVFNVTLYY*	12*∗*	49
*LYYKHITTV*	6	21
*TTVTTWALF*	6	16
*MATGDTVEI*	6	0
*DTVEISPFY*	5	5
*YRFLEIANY*	6	49
*VSYKNSYHF*	9*∗*	15
*YKNSYHFSL*	5	45
*HVRSGDIEY*	5	0
*MSHGLAEMY*	6	22
*VVMTAAAAV*	5	42
*FAYDKIQAH*	5	49
*YLLYEDYTF*	9*∗*	48
*IASFLSNPF*	6	67^#^
*FLSNPFAAL*	8	58
*KSNPVQVLF*	5	0
*LLGDIVAVS*	0	60
*FLTDEQFTI*	3	76^#^
*FLEIANYQV*	2	67^#^
*LLRSTVSKA*	0	64

*∗*Proposed MHCI docked epitopes; ^#^top proposed MHCII epitopes.

**Table 6:** The binding energy and attractive VDW scores for the suggested epitopes with chicken BF2 alleles using PatchDock server.

Ligand	Receptor	Global energy kcal/mol	Attractive VDW kcal/mol
YVFNVTLYY	BF_2_ 2101	-91.78	-32.23
	BF_2_ 0401	- 45.65	-27.43

YLLYEDYTF	BF_2_ 2101	-89.53	-34.81
	BF_2_ 0401	-61.68	-27.77

VSYKNSYHF	BF_2_ 2101	-66.41	-28.95
	BF_2_ 0401	-51.56	-30.71

## Data Availability

The sequences of envelope glycoprotein B (GB) of ILTV were retrieved from GenBank of National Center for Biotechnology Information (NCBI) (http://www.ncbi.nlm.nih.gov/protein) in August 2017. Retrieved strains and their accession numbers and geographical regions were listed in Table 1.
